# Placental monoamine oxidase A activity in pregnancies complicated by maternal overweight/obesity and gestational diabetes mellitus

**DOI:** 10.3389/fendo.2026.1728858

**Published:** 2026-03-13

**Authors:** Maja Perić, Marina Horvatiček, Kristina Nikolić, Lipa Čičin-Šain, Jasminka Štefulj

**Affiliations:** 1 Division of Molecular Biology, Ruđer Bošković Institute, Zagreb, Croatia; 2 Department of Biology, Faculty of Science, Zagreb, Croatia

**Keywords:** GDM, MAO, obesity, overweight, placenta, pregnancy, serotonin, enzyme kinetics

## Abstract

**Introduction:**

The human placenta is a major site of metabolic transformation, synthesizing and catabolizing a wide range of bioactive compounds and thereby contributing to pregnancy success. Monoamine oxidase (MAO) is an important component of placental catabolic systems, catalyzing the oxidative deamination of biogenic monoamines, including serotonin and catecholamines.

**Methods:**

We developed a high-throughput fluorimetric assay using six concentrations of kynuramine as substrate to determine the kinetic parameters - maximum velocity (V_max_) and Michaelis affinity constant (K_m_) - of MAO activity in human placental tissue. Pharmacological experiments with selective MAO-A and MAO-B inhibitors identified MAO-A as the sole catalytically active MAO isoform in human term placenta. We applied the assay to placental samples from 93 women to assess whether maternal overweight/obesity (OWO) and/or gestational diabetes mellitus (GDM) are associated with changes in placental MAO-A kinetic parameters, and to examine the relationship between placental *MAOA* mRNA levels and MAO-A catalytic capacity.

**Results:**

Maternal OWO was not associated with changes in V_max_ or K_m_, nor was GDM associated with changes in Vmax (all p>0.05). However, GDM was associated with a modest increase in K_m_ (*p*=0.024), indicating reduced substrate affinity. In metabolically healthy pregnancies, placental MAOA mRNA levels correlated positively with V_max_ (rp=0.68, p=0.001), while this relationship was absent in placentas from women with OWO and/or GDM (*p*>0.05).

**Conclusions:**

Our findings suggest no alterations in placental monoamine catabolism in pregnancies complicated by maternal OWO, but indicate possible subtle changes in those complicated by GDM. The positive correlation between placental *MAOA* expression and MAO-A catalytic capacity in metabolically healthy pregnancies supports the use of *MAOA* mRNA levels as a proxy for MAO-A catalytic activity under physiological conditions. However, metabolic disturbances may disrupt this coupling, underscoring the value of the standardized kinetic assay described here as a robust tool for future studies of placental MAO-A function.

## 1 Introduction

The human placenta, the maternal–fetal interface essential for a successful pregnancy, is a highly metabolically active organ (1). In addition to synthesizing hormones and cytokines that regulate maternal physiology and fetal development, the placenta actively catabolizes numerous bioactive substances, thereby maintaining their optimal levels at the maternal–fetal interface and in maternal and fetal circulation. Among these substances are the biogenic monoamines serotonin, dopamine, epinephrine, and norepinephrine (2–4), which are potent signaling molecules that modulate multiple physiological processes, with serotonin playing a particularly important role in regulating placental steroidogenesis, nutrient transport, and both placental and fetal development (5–7).

Monoamine oxidase (MAO), a flavoprotein located on the outer mitochondrial membrane, is the principal enzyme responsible for biogenic monoamine catabolism, catalyzing oxidative deamination and thus contributing to monoamine signaling homeostasis and xenobiotic monoamine degradation (8). It exists in two isoforms, MAO-A and MAO-B, encoded by distinct genes on the X chromosome, *MAOA* and *MAOB*, respectively. The isoforms share substantial structural homology, but differ in substrate affinity (with MAO-A favoring serotonin and norepinephrine) and tissue distribution (9). In the human placenta, *MAOA* is abundantly expressed in syncytiotrophoblasts, cytotrophoblasts, and feto-placental endothelial cells (10–12), while *MAOB* expression is minimal or undetectable (13–16), establishing MAO-A as the key isoform in this organ.

Several studies have examined associations between pregnancy complications and placental *MAOA* expression. Elevated maternal depressive symptoms during pregnancy have been associated with decreased placental *MAOA* mRNA levels (17), while preeclampsia has been associated with increased placental *MAOA* mRNA and protein levels (18), although not consistently (19). In addition, several studies have reported reduced MAO-A catalytic activity in placentas from pregnancies complicated by preeclampsia, hypertension, or diabetes mellitus (19–21). Given the central role of MAO-A in placental monoamine catabolism, changes in placental *MAOA* expression or MAO-A catalytic activity may affect placental development and function, with potential long-term consequences for offspring outcomes. Supporting this, placental *MAOA* mRNA levels have been shown to mediate the association between maternal stress during pregnancy and specific aspects of offspring temperament at 12 months of age (22). These findings underscore the importance of better understanding changes in placental *MAOA* regulation in the context of pregnancy complications.

Maternal overweight/obesity (OWO) and gestational diabetes mellitus (GDM) are increasingly prevalent pregnancy complications with significant adverse effects on both maternal and offspring health (23, 24). Both conditions are associated with placental inflammation, oxidative stress, and metabolic alterations (25, 26). However, despite their well-documented impact on the placenta, evidence regarding placental *MAOA* regulation in these conditions is limited. An *in vitro* study showed that high glucose, a hallmark of GDM, reduces *MAOA* mRNA levels in a first-trimester trophoblast cell line (27). Another study found that maternal OWO and GDM were not associated with *MAOA* mRNA levels in term placentas (28). To date, no study has investigated placental MAO-A catalytic activity in the context of maternal OWO and/or GDM.

In this study, we developed a robust, high-throughput, microplate-based assay to determine the kinetic parameters of MAO-A activity in human term placenta. Using this method, we investigated whether maternal OWO and/or GDM are associated with alterations in placental MAO-A kinetic parameters. In addition, we examined the relationship between placental *MAOA* mRNA levels and MAO-A catalytic capacity. Together, these analyses provide new insights into placental monoamine catabolism in pregnancies complicated by maternal metabolic disorders.

## 2 Materials, subjects and methods

### 2.1 Participants

Women included in the study were recruited at the Department of Gynecology and Obstetrics, University Hospital Centre Zagreb, as part of the PlaNS (Placental and Neonatal Serotonin) cohort study (project code: IP−2018-01-6547; initiated December 1, 2018). Inclusion criteria for the PlaNS cohort were planned cesarean section, absence of diabetes prior to pregnancy, and absence of preeclampsia or other life-threatening conditions. Demographic and clinical data were obtained from medical records and questionnaires, as previously described (28–30). Pre-pregnancy body mass index (BMI) was calculated as pre-pregnancy body weight (kg) divided by height squared (m^2^). Normal weight (NW) was defined as BMI of 18.0 to 24.9 kg/m^2^, and OWO as BMI ≥ 25.0 kg/m^2^. Women were classified as having GDM based on the diagnostic criteria of the International Association of Diabetes and Pregnancy Study Groups (IADPSG) (31, 32); those not meeting these criteria were classified as having normal glucose tolerance (NGT). Gestational age was determined based on the first day of the last menstrual period and adjusted if necessary (33).

For the present study, inclusion criteria were singleton pregnancies, full-term gestational age (≥37 weeks), normal birth weight (2500–4499 g), and availability of placental tissue for MAO-A activity analysis. Exclusion criteria were suspected or diagnosed intrauterine growth restriction, macrosomia, congenital anomalies, and use of MAO inhibitors or any class of antidepressants during pregnancy. From the eligible pool, 93 participants were selected to ensure balanced representation in four metabolic groups defined by BMI status before pregnancy and glucose tolerance status during pregnancy (i.e., NW and NGT, NW and GDM, OWO and NGT, and OWO and GDM).

The study was approved by the Ethics Committee of University Clinical Hospital Centre Zagreb (class: 8.1-18/162-2, number: 02/21 AG; approved on 18.07.2018) and the Bioethics Committee of the Ruđer Bošković Institute, Zagreb (BEP-8761/2-2018; approved on 26.11.2018). All participants gave written informed consent to participate in the study. All procedures were conducted in accordance with the Declaration of Helsinki.

### 2.2 Placental and platelet samples

Placental tissue was collected within 5 minutes after birth, using a standardized sampling procedure described previously (34). Briefly, tissue pieces were excised from the fetal side of the placenta (2–3 pieces per placental quadrant), pooled, snap-frozen, and stored at –80 °C until further processing. For protein extraction, 50 mg of tissue was homogenized in 0.05 M potassium phosphate (KH_2_PO_4_) buffer (pH 7.6) at a ratio of 1:20 (tissue mass in g to buffer volume in mL) by sonication (3 cycles of 10 seconds at 20 kHz, amplitude 8 µm; B. Braun Biotech International, Germany). Samples were kept on ice throughout to prevent protease activity. The homogenates were centrifuged at 2000 × g for 5 minutes at 4 °C and the supernatants were stored at –80 °C until further processing. Total protein concentrations in supernatants were quantified using the Pierce BCA Protein Assay Kit (Thermo Fisher Scientific Inc., Foster City, CA, USA) according to the manufacturer’s instructions.

Blood samples for platelet preparation were collected in ACD-A tubes (VACUETTE^®^ TUBE 9 mL, Greiner Bio-One, Kremsmünster, Austria) and gently inverted several times to ensure proper mixing with the anticoagulant. Platelet-rich plasma (PRP) was obtained by centrifugation at 1200 × g for 2 minutes. Platelet counts in PRP were determined using the DxH 500 hematology analyzer (Beckman Coulter, Brea, CA, USA). PRP was diluted 1:5 with physiological solution and centrifuged at 4500 × g for 7 minutes at 4 °C to pellet the platelets. Platelet pellets were homogenized in 0.05 M KH_2_PO_4_ (pH 7.6; volume equal to the initial PRP volume) by sonication (3 × 10 seconds, 20 kHz, amplitude 8 µm). MAO activity assays were performed using 20 x 10^6^ platelets per reaction, as adapted from our previous study (35).

### 2.3 MAO activity assay

MAO activity in human term placenta was measured using a fluorimetric assay with kynuramine (3-[2-aminophenyl]-3-oxopropanamine, Sigma-Aldrich, St. Louis, MO, USA) as the substrate, based on a platelet MAO activity procedure previously validated in our laboratory (35). For this study, the assay was adapted to a 96-well microplate format with a total reaction volume of 180 µl. The reaction mixture comprised placental protein, kynuramine, and, when applicable, inhibitor solutions, all prepared in 0.033 M sodium borate buffer (pH 8.3). Placental protein dilutions were prepared on ice, distributed into the microplate, and preincubated for 10 minutes at 37 °C before substrate addition. Reactions were carried out at 37 °C and terminated by deproteinization with 1 M trichloroacetic acid (TCA; 90 μL per reaction). For each sample, a corresponding blank deproteinized prior to substrate addition was processed in parallel. Each microplate also included six concentrations of the reaction product 4-hydroxyquinoline (4-hydroxy-1-azanaphthalene, Sigma-Aldrich, St. Louis, MO, USA) to generate a standard curve for product quantification, as well as a reference sample to monitor interassay variability. Fluorescence was measured at 310 nm excitation and 362 nm emission using an Infinite 200 PRO multimodal microplate reader (Tecan Austria GmbH, Grödig, Austria). Optimal assay conditions for placental samples were determined in preliminary experiments with varying placental protein amounts (1.5, 3.0, 6.0, 12.0, 24.0 and 48.0 μg per reaction), substrate concentrations (10, 30 and 100 µM), and incubation times (10, 20 and 30 minutes) (see Results section). Based on the preliminary experiments, all subsequent assays were performed using 6 µg of placental protein and a 15-minute incubation.

To assess the relative contributions of MAO-A and MAO-B isoforms to total placental MAO activity, we performed pharmacological experiments using the selective MAO-A inhibitor clorgyline (N-methyl-N-propargyl-3-(2,4-dichlorophenoxy) propylamine hydrochloride, Sigma-Aldrich, St. Louis, MO, USA), and the selective MAO-B inhibitor deprenyl (selegiline; R- (–)-deprenyl hydrochloride, Research Biochemicals International, Natick, MA, USA). Previous reports show that clorgyline selectively inhibits MAO-A and deprenyl selectively inhibits MAO-B at nanomolar concentrations (36–38). Clorgyline was used at final concentrations of 10–^9^ M and 10–^6^ M. Deprenyl was used at final concentrations ranging from 10–^9^ M to 10–^5^ M. Before adding the kynuramine substrate at a final concentration of 50 µM, samples were preincubated with inhibitors for 10 minutes at 37 °C. Human platelets, which contain only the MAO-B isoform (39), were included as controls in these experiments.

Kinetic parameters – the maximum velocity (V_max_, nmol/min/mg protein), representing the maximal reaction rate under saturating substrate conditions and reflecting the amount of catalytically active enzyme, and the Michaelis constant (K_m_, µM), reflecting the apparent enzyme-substrate affinity – were determined by measuring reaction velocities at six kynuramine concentrations (6.25, 12.5, 25, 50, 100 and 200 µM). Placental samples from the four metabolic groups were evenly distributed across microplates. The measured reaction velocities were plotted against substrate concentrations and fitted to the Michaelis-Menten kinetic model using GraphPad Prism v.8 (GraphPad Software, LLC, San Diego, CA, USA). V_max_ and K_m_ values were estimated by nonlinear least-squares regression analysis.

### 2.4 Statistical analyses

Statistical analyses were performed using GraphPad Prism v.8 (GraphPad Software Inc., San Diego, CA, USA). Outliers were identified using the Robust Regression and Outlier Removal (ROUT) method with a Q value (maximum false discovery rate) set at 1% (40) or the Grubbs method (41). The normality of data distribution was assessed using the D’Agostino-Pearson test. Continuous variables were compared among groups using one-way ANOVA with Tukey’s *post-hoc* test or the Kruskal–Wallis test with Dunn’s *post-hoc* test, depending on data normality. The chi-square test was used to compare frequency distributions among groups. The interaction effect of maternal pregestational body weight status and gestational glucose tolerance status on MAO-A kinetic parameters was tested using two-way ANOVA. Group means were estimated as least squares (LS) means. Correlation analyses were performed using Pearson’s or Spearman’s correlation test, depending on data normality. All statistical tests were two-sided, with the significance level set at *p ≤* 0.05.

## 3 Results

### 3.1 Characteristics of the study sample

Demographic and clinical characteristics of the study participants, classified by BMI status before pregnancy and glucose tolerance status during pregnancy into four metabolic groups – normal weight with normal glucose tolerance (NW-NGT), normal weight with gestational diabetes mellitus (NW-GDM), overweight/obesity with normal glucose tolerance (OWO-NGT), and overweight/obesity with gestational diabetes mellitus (OWO-GDM) – are summarized in **Table 1**. Maternal age, parity, smoking during pregnancy, newborn sex, birth weight, and birth length did not differ significantly among the four metabolic groups, while gestational age at childbirth, pre-pregnancy BMI, and gestational weight gain showed significant overall differences (**Table 1**). *Post-hoc* comparisons showed no significant pairwise differences in gestational age (all *p*>0.05). As expected, pre-pregnancy BMI was significantly higher in women with OWO than in women with NW, regardless of glucose tolerance status (all *p* < 0.0001). Gestational weight gain was higher in the NW-NGT group than in the OWO-NGT group (*p* < 0.0001), while no difference was observed between the NW-GDM and OWO-GDM groups (*p*>0.05). Likewise, no significant pairwise differences in pre-pregnancy BMI or gestational weight gain were observed between the NW-NGT and NW-GDM groups or between the OWO-NGT and OWO-GDM groups (all *p*>0.05).

**Table 1:** Demographic and clinical characteristics of study participants stratified by metabolic status.

Characteristics	NW-NGT	NW-GDM	OWO-NGT	OWO-GDM	*p*-value
Number of participants	21	30	21	21	
Maternal age, years	34.1 [31.9 – 38.0]	34.6 [31.2 – 39.2]	32.2 [29.0 – 36.0]	35.5 [31.5 – 38.7]	0.831 ^a^
Gestational age, weeks	39.3 [39.0 – 39.8]	39.4 [39.0 – 39.9]	39.3 [38.9 – 39.8]	38.7 [38.0 – 39.4]	**0.035** ^a^
Pre-pregnancy BMI, kg/m^2^	21.3 [20.1 – 22.9]	22.6 [21.6 – 23.4]	31.7 [26.5 – 33.5]	28.0 [25.9 – 29.5]	**<0.0001** ^b^
Gestational weight gain, kg	17.0 [14.0 – 19.0]	12.3 [9.8 – 15.0]	10.0 [7.5 – 14.0]	12.0 [8.0 – 16.8]	**0.0003** ^b^
Primiparity, n (%)	7 (33.3)	14 (46.7)	6 (28.6)	9 (42.9)	0.548 ^c^
Smoking in pregnancy, n (%)^d^	4 (19.0)	5 (17.9)	6 (28.6)	6 (28.6)	0.721 ^c^
Male newborn, n (%)	7 (33.3)	16 (53.3)	13 (61.9)	10 (47.6)	0.296 ^c^
Birth weight, g	3410 [3160 – 3590]	3405 [3168 – 3740]	3520 [3355 – 3745]	3630 [3290 – 3855]	0.334 ^a^
Birth length, cm	49 [49 – 52]	50 [48 – 51]	50 [49 – 51]	49 [49 – 51]	0.839 ^a^

Continuous variables are presented as median [interquartile range], and categorical variables as number of subjects (n) and percentage (%). Group differences were assessed using ^a^one-way ANOVA, ^b^Kruskal-Wallis test, or ^c^Chi-square test, as appropriate. Statistically significant *p*-values are shown in bold. ^d^Women who smoked during pregnancy or quit after conception were classified as smokers, while those who never smoked or quit 6 or more months before pregnancy were classified as non-smokers, with unclear cases (n=2) treated as missing data. NW-NGT, normal weight and normal glucose tolerance; NW-GDM, normal weight and gestational diabetes mellitus; OWO-NGT, overweight/obesity and normal glucose tolerance; OWO-GDM, overweight/obesity and gestational diabetes mellitus; BMI, body mass index.

### 3.2 Optimization of the MAO activity assay

**Figure 1:**
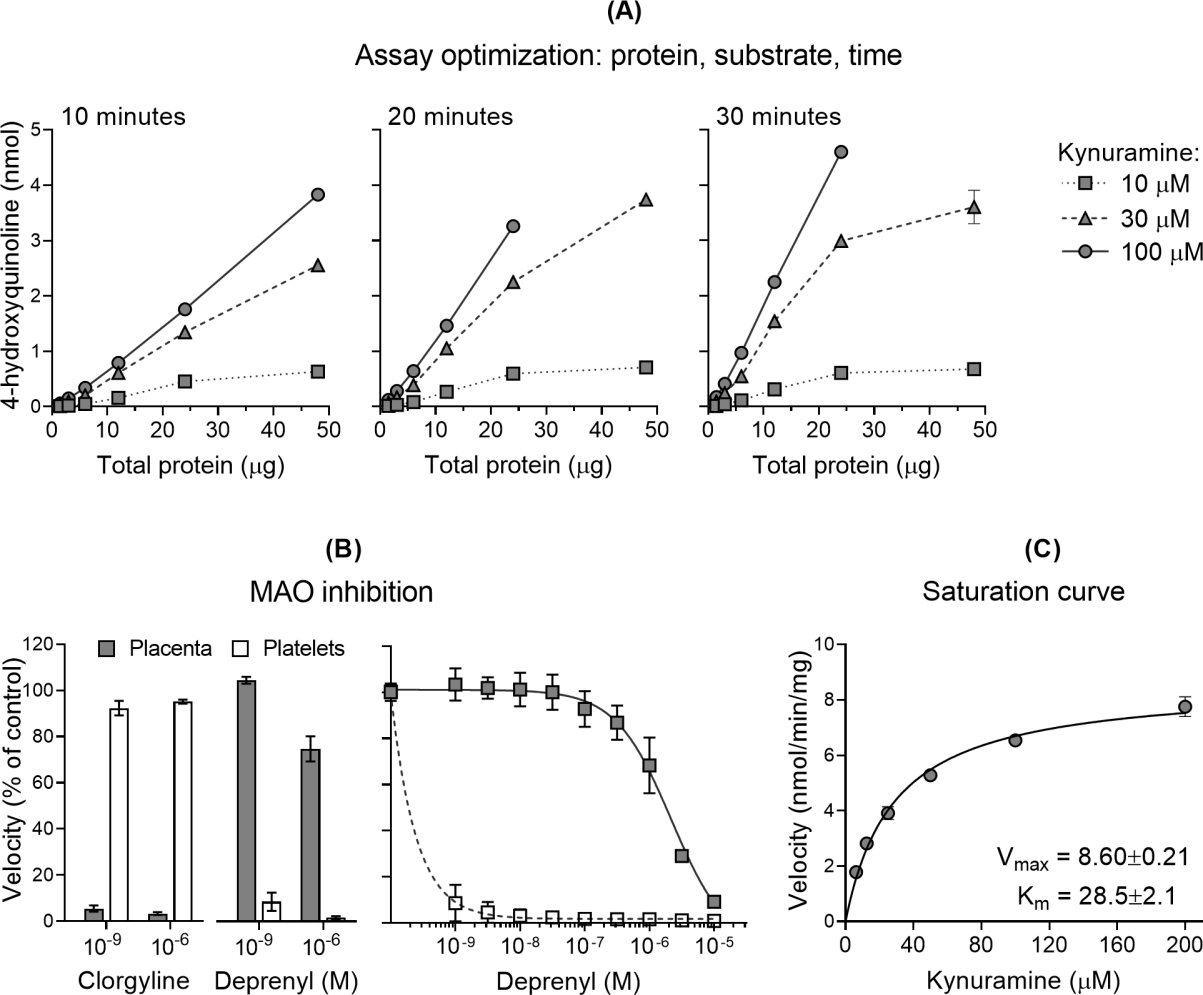
Optimization of placental monoamine oxidase (MAO) activity assay. **(A)** Formation of 4-hydroxyquinoline as a function of placental protein amount, kynuramine (substrate) concentration, and incubation time. Reactions were performed with varying total protein amounts (0.0, 1.5, 3.0, 6.0, 12.0, 24.0, and 48.0 µg), kynuramine concentrations (10, 30, and 100 µM), and incubation times (10, 20 and 30 minutes). Values are shown as means ± standard deviations (n=2). **(B)** Effect of the MAO-A inhibitor clorgyline and the MAO-B inhibitor deprenyl on 4-hydroxyquinoline formation in placental (n=4-6) and platelet (n=2) samples. Kynuramine concentration was 50 µM. Values are expressed as a percentage of control and presented as means ± standard deviations. [**(B)** left panel] Clorgyline and deprenyl were applied at concentrations of 10–^9^ and 10–^6^ M [**(B)** right panel] Deprenyl was applied at concentrations from 10–^9^ to 10–^5^ M (log scale). Values were fitted to non-linear regression curves. **(C)** Kinetic analysis of placental MAO activity. Reactions were performed with 6 µg placental protein at six kynuramine concentrations (6.25, 12.5, 25, 50, 100, and 200 µM) during a 15-minute incubation at 37 °C. Reaction velocities, calculated from product formation, were plotted against substrate concentration and fitted to the Michaelis–Menten kinetic model. V_max_ and K_m_ values were estimated by nonlinear least-squares regression analysis. Data from a representative sample are shown (mean ± standard error of the mean; n=3).

Preliminary experiments varying placental protein amounts, incubation times, and substrate (kynuramine) concentrations showed that formation of the reaction product 4-hydroxyquinoline increased linearly with protein amounts ranging from 1.5 to 24 µg per reaction under all conditions tested (**Figure 1A**). Based on these findings, 6 µg of total protein and a 15-minute incubation were selected for subsequent experiments.

We next assessed the contribution of MAO isoforms to 4-hydroxyquinoline formation in placental protein extracts, using pharmacological inhibitors. The selective MAO-A inhibitor clorgyline, at both nanomolar and micromolar concentrations, reduced 4-hydroxyquinoline formation in placental samples to less than 5% of control levels, while showing no effect in platelets, which contain only the MAO-B isoform (**Figure 1B**, left panel). In contrast, the MAO-B inhibitor deprenyl at nanomolar concentrations strongly inhibited 4-hydroxyquinoline formation in platelets, consistent with selective MAO-B inhibition at low concentrations, while placental samples were unaffected by deprenyl at concentrations up to the micromolar range (half-maximum inhibitory concentration, IC_50_ = 4.18×10–^6^ M; **Figure 1B**, right panel), indicating negligible MAO-B activity in human placenta. These findings demonstrate that 4-hydroxyquinoline formation in placental samples is attributable to MAO-A, eliminating the need for selective inhibitors in subsequent kinetic assays.

Kinetic assays measuring reaction velocities at six substrate concentrations (6.25 to 200 µM) produced clear saturation curves consistent with Michaelis-Menten enzyme kinetics, enabling reliable estimation of the kinetic parameters V_max_, which reflects the total amount of catalytically active enzyme, and K_m_, which reflects the apparent enzyme-substrate affinity (**Figure 1C**).

### 3.3 Kinetic parameters of placental MAO activity in relation to maternal OWO and GDM

**Figure 2:**
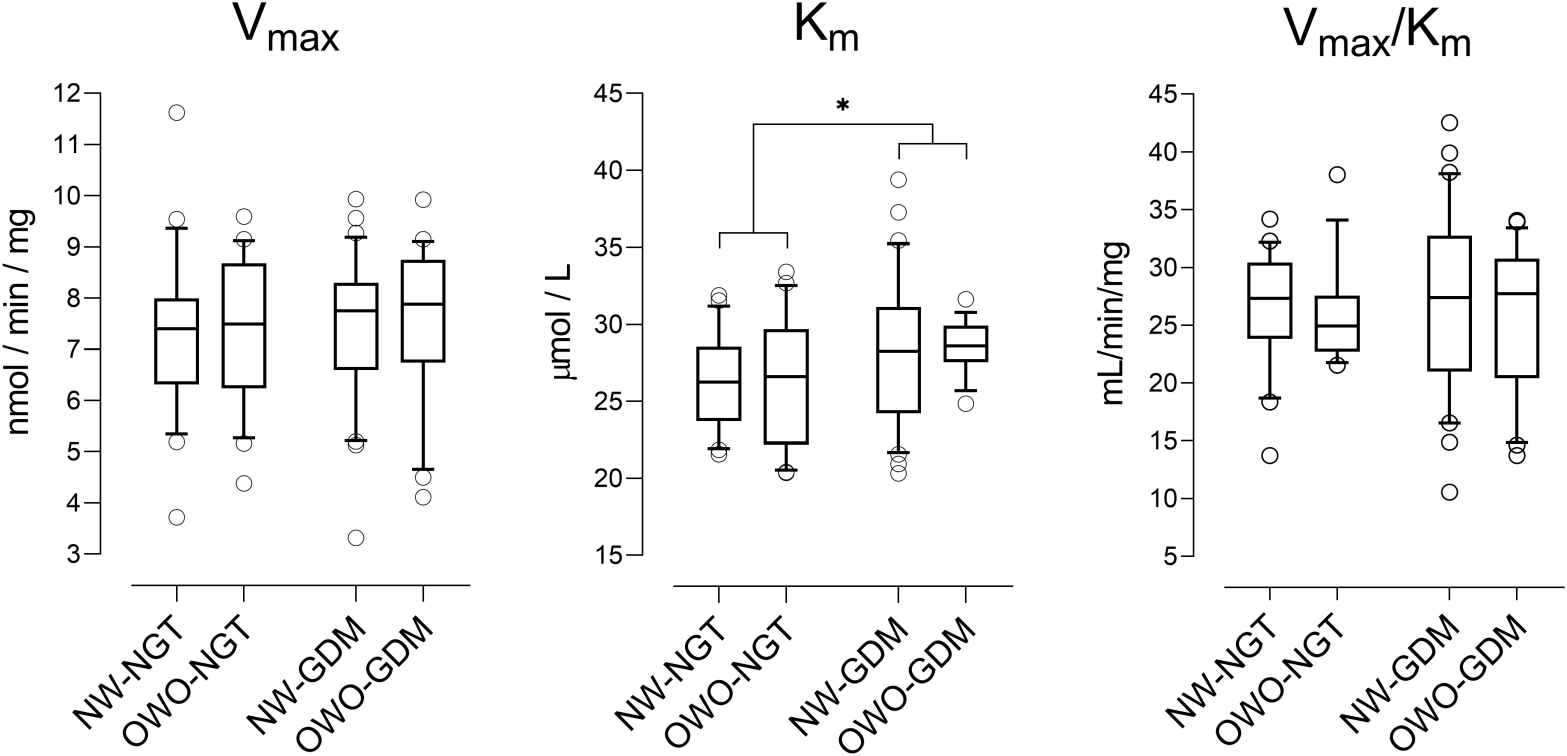
Kinetic parameters of placental MAO-A in relation to maternal metabolic status. Kinetic parameters V_max_ and K_m_ were determined from Michaelis-Menten saturation curves (Appendix Figure A1), as illustrated in **Figure 1C**. Data for women with normal weight and normal glucose tolerance (NW-NGT; n=21), overweight/obesity and normal glucose tolerance (OWO-NGT, n=21), normal weight and gestational diabetes mellitus (NW-GDM, n=30), and overweight/obesity and gestational diabetes mellitus (OWO-GDM, n=21) are presented as boxplots showing the median and interquartile range, with whiskers representing the 10th to 90th percentiles and open circles indicating individual values outside the whisker range. V_max_, maximum velocity; K_m_, Michaelis constant; V_max_/K_m_, catalytic efficiency. *p < 0.05 for the main effect of glucose tolerance status (two-way ANOVA).

**Figure A1:**
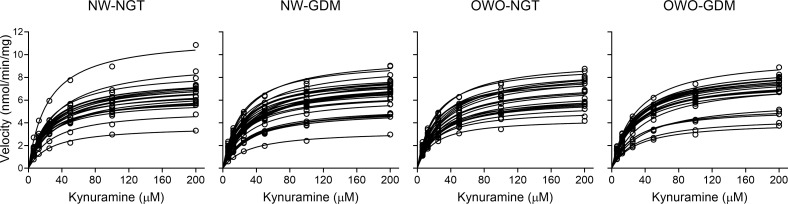
Michaelis–Menten kinetics curves for MAO activity in placental samples from study participants (n=93). Reactions were performed with 6 µg total placental protein, at six concentrations of the substrate kynuramine (6.25, 12.5, 25, 50, 100, and 200 µM), during a 15-minute incubation at 37 °C. Reaction velocities were calculated from product formation, plotted as a function of substrate concentration, and fitted to the Michaelis–Menten kinetic model. Shown are curves for women with normal weight and normal glucose tolerance (NW-NGT, n=21), normal weight and gestational diabetes mellitus (NW-GDM, n=30), overweight/obesity and normal glucose tolerance (OWO-NGT, n=21), and overweight/obesity and gestational diabetes mellitus (OWO-GDM, n=21).

Placental MAO-A kinetic parameters (V_max_ and K_m_) were estimated from substrate saturation curves for all study participants (Appendix Figure A1) and compared among the metabolic groups (**Figure 2**). In placentas from metabolically healthy pregnancies (NW-NGT; n=21), V_max_ was 7.26 ± 1.60 nmol/min/mg protein (mean ± SD; range 3.72 to 11.62), and K_m_ was 26.4 ± 3.0 µM (mean ± SD; range 21.6 to 31.9). V_max_ did not differ by maternal body weight status (*p* = 0.573) or glucose tolerance status (*p* = 0.599). Similarly, K_m_ did not differ by maternal body weight status (*p* = 0.746), but was slightly higher in women with GDM (LS mean 28.4 µM) compared to women with NGT (LS mean 26.5 µM; *p* = 0.024), indicating a modest reduction in substrate affinity of placental MAO-A in women with GDM. Catalytic efficiency (V_max_/K_m_) did not differ by maternal body weight status (*p* = 0.849) or glucose tolerance status (*p* = 0.570).

### 3.4 Correlation between placental *MAOA* mRNA levels and V_max_

**Figure 3:**
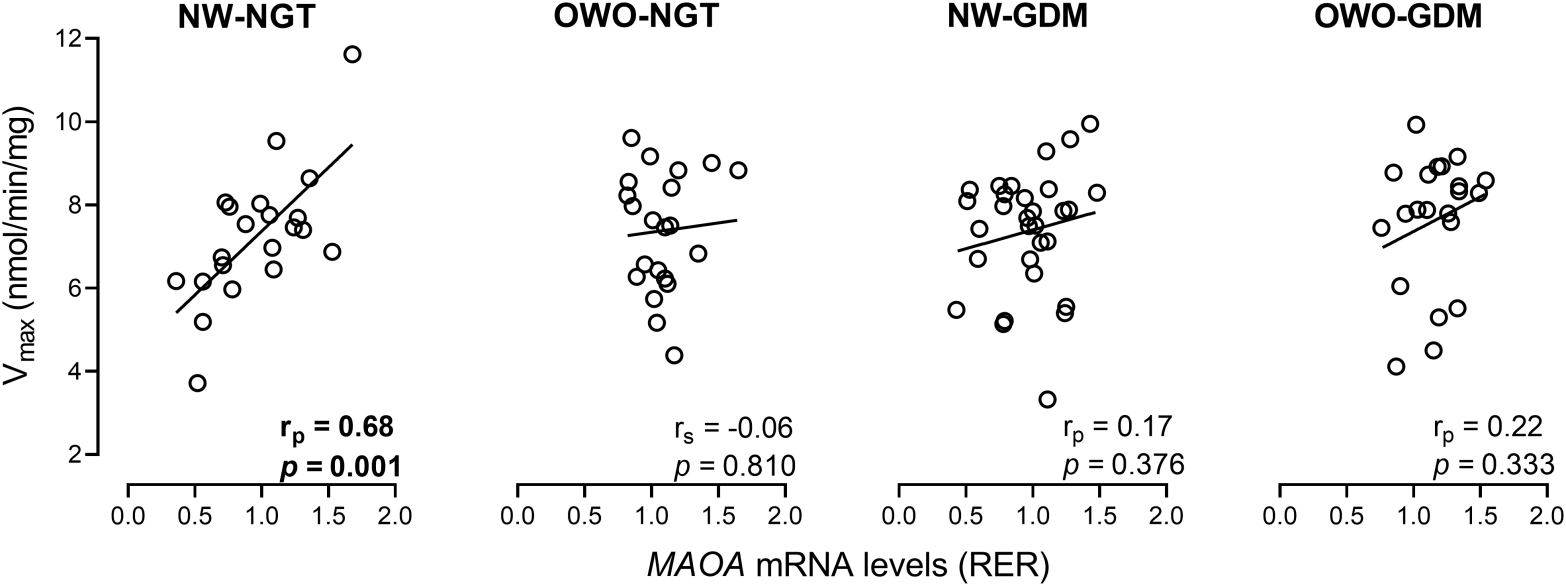
Correlation between placental *MAOA* mRNA levels and maximum velocity (V_max_) values of placental MAO-A in women with normal weight and normal glucose tolerance (NW-NGT), normal weight and gestational diabetes mellitus (NW-GDM), overweight/obesity and normal glucose tolerance (OWO-NGT), and overweight/obesity and gestational diabetes mellitus (OWO-GDM). *MAOA* mRNA levels were normalized to *YWHAZ* mRNA levels (28). r_p_, Pearson’s correlation coefficient; RER, relative expression ratio.

**Figure A2:**
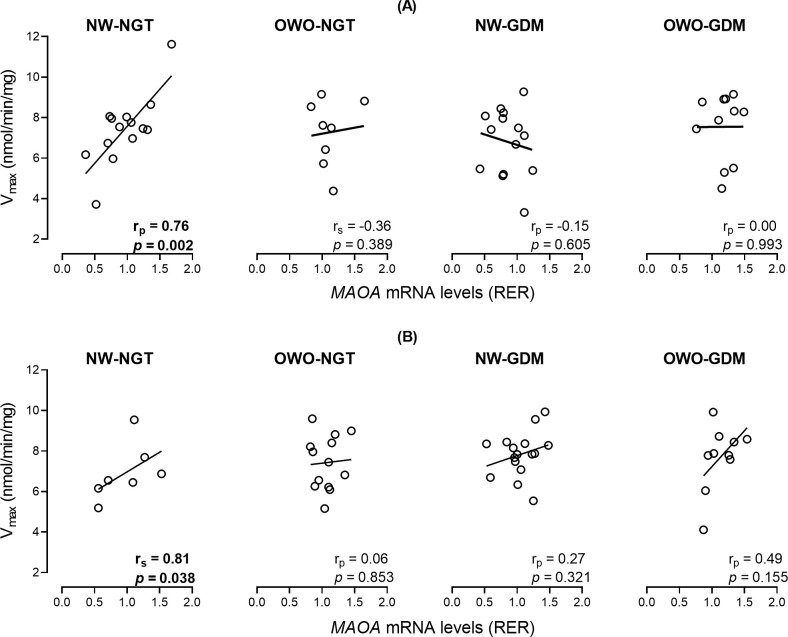
Correlation between placental *MAOA* mRNA levels and maximum velocity (V_max_) values of placental MAO-A in women with normal weight and normal glucose tolerance (NW-NGT), normal weight and gestational diabetes mellitus (NW-GDM), overweight/obesity and normal glucose tolerance (OWO-NGT), and overweight/obesity and gestational diabetes mellitus (OWO-GDM). (A) Female newborns, n=48; (B) male newborns, n=47. The *MAOA* mRNA levels were normalized to YWHAZ mRNA levels (28). r_p_, Pearson’s correlation coefficient; r_s_, Spearman’s correlation coefficient; RER, relative expression ratio.

We next examined whether placental *MAOA* mRNA levels reflect MAO-A catalytic capacity (as represented by V_max_) by correlating placental *MAOA* expression data from our previous study (28) with V_max_ values obtained here. A strong positive correlation was observed in the NW-NGT group (r_p_=0.68, *p* = 0.001), while no significant correlations (all *p*>0.05) were detected in the OWO-NGT, NW-GDM and OWO-GDM groups (**Figure 3**), or when all metabolically complicated pregnancies were analyzed together to increase statistical power (n=72, r_p_= 0.17, *p* = 0.157). Comparable results were obtained in sex-specific sensitivity analyses (Appendix Figure A2). Together, these findings indicate a clear positive association between placental *MAOA* expression levels and MAO-A catalytic capacity in metabolically healthy pregnancies – an association absent in pregnancies complicated by maternal OWO and/or GDM.

## 4 Discussion

In this study, we developed and validated a simple microplate-based fluorimetric assay for the reliable determination of kinetic parameters (V_max_ and K_m_) of the MAO-catalyzed reaction in human term placenta. We optimized key assay conditions, including protein concentration, incubation time, and substrate range, enabling robust kinetic analysis with higher throughput compared to radiotracer-based (14, 19, 20) or HPLC-based methods (11, 21, 42). Experimental conditions such as reaction temperature, incubation time, sample type (whole tissue homogenate, mitochondrial homogenate, intact mitochondria), buffer, pH, and the presence of inhibitors or detergents influence enzyme activity and may affect apparent kinetic parameters, while the choice of detection method (radiometric, spectrophotometric, fluorimetric, or HPLC-based measurement of product) influences assay sensitivity and linearity. These considerations underscore the importance of standardized kinetic assays using a range of substrate concentrations spanning the K_m_ to ensure reproducible measurements and comparable estimates of MAO-A activity across studies.

We used kynuramine as a model substrate, as it is oxidatively deaminated by both MAO-A and MAO-B isoforms. Using the selective MAO-A and MAO-B inhibitors clorgyline and deprenyl, respectively, we showed that placental MAO activity is exclusively attributable to the MAO-A isoform. An earlier study reported detectable, albeit low, MAO-B protein and activity levels in the human placenta (15). However, our finding is consistent with most previous studies employing pharmacological (14, 16, 20), immunohistochemical (19) and western blot (21) analyses, supporting the consensus that MAO-A is the predominant isoform responsible for oxidative deamination in the human placenta.

Using the optimized protocol, we characterized kinetic parameters of placental MAO-A activity in metabolically healthy pregnancies and pregnancies complicated by maternal OWO and/or GDM. The K_m_ values we obtained in healthy pregnancies were within the lower micromolar range, consistent with prior reports using kynuramine in a multi-point kinetic analyses (43, 44). V_max_ values depend strongly on enzyme source and assay conditions and are therefore not directly comparable across studies.

A key novel finding of our work is that maternal OWO was not associated with changes in placental MAO-A kinetics, while GDM was linked to a modest increase in K_m_, without alterations in V_max_. These results indicate that maternal adiposity alone does not affect placental monoamine catabolism, while GDM may reduce MAO-A substrate affinity. To our knowledge, no prior study has investigated placental MAO-A activity in the context of maternal OWO or GDM. An earlier study reported reduced MAO activity in placentas from women with pre-existing diabetes, but it relied on single-concentration measurements (20), limiting direct comparison with our multi-point kinetic analysis.

The possible mechanisms underlying the observed increase in K_m_ under GDM conditions, reflecting reduced affinity of placental MAO-A for its substrate are unclear but may involve GDM-associated oxidative, metabolic, inflammatory, or hormonal changes in the placental milieu that could selectively affect enzyme-substrate interactions without altering V_max_ (25, 26). Such changes could potentially induce post-translational modifications of MAO-A or affect its mitochondrial membrane environment, thereby impairing enzyme - substrate interactions. The accumulation of certain lipids in the membrane, for instance, may increase macromolecular crowding, limiting substrate access and effectively lowering substrate affinity without altering maximum velocity (45, 46). Lipidomic studies show marked alterations in GDM placentas (47, 48), which could potentially drive such effects. Supporting this, reduced placental MAO-A activity has been reported in preeclampsia and hypertensive pregnancies (19–21), conditions also characterized by metabolic stress. However, direct evidence connecting the proposed mechanisms with changes in MAO-A catalytic parameters is lacking, highlighting a substantial gap in current research and underscoring the need for targeted *in vitro* studies addressing MAO-A enzyme regulation under conditions of metabolic stress.

Although changes observed in K_m_ were modest, they may still be relevant for placental MAO-A activity. Placental MAO-A is crucial for inactivation of serotonin and catecholamines at the maternal-fetal interface, which are potent vasoactive mediators influencing uteroplacental and umbilical vascular tone (49–51). In addition, serotonin influences placental development (52, 53), hormone production (54, 55) and nutrient transfer (56), and is essential for histone serotonylation, a modification that regulates the expression of numerous placental genes (6, 57). Serotonin of placental or maternal origin also supports proper fetal brain development (6, 7). Placental MAO-A contributes to serotonin clearance from the fetal circulation at term (42, 58), while placental *MAOA* mRNA levels have been implicated in mediating the effects of prenatal stress on infant temperament (22). Taken together, the subtle alterations in placental MAO-A kinetics could modify biogenic monoamine availability, with possible downstream consequences for placental function, fetal development, and long-term offspring health.

Our findings show that placental *MAOA* mRNA levels correlate positively with MAO-A catalytic capacity (V_max_) in metabolically healthy pregnancies, suggesting that *MAOA* transcript levels may serve as a valid proxy for MAO-A catalytic activity in placentas under physiological conditions. However, this relationship is lost in pregnancies complicated by maternal OWO and/or GDM, indicating that maternal metabolic disturbances associated with OWO and/or GDM may impair the normal coupling between *MAOA* expression and MAO-A catalytic activity, possibly by affecting post-transcriptional and post-translational regulatory pathways or through mitochondrial membrane changes that affect MAO-A catalytic capacity. Several previous studies measured both *MAOA* mRNA levels and MAO-A enzymatic activity in human placentas (11, 19, 42), but did not formally examine their intercorrelations. Nevertheless, these studies reported discrepancies between mRNA and activity findings, such as reduced MAO-A activity in preeclamptic placentas despite unchanged *MAOA* mRNA levels (19), or even opposite mRNA and activity trends when comparing first-trimester and term placentas (11), supporting the notion that *MAOA* expression does not necessarily predict functional enzyme output. Taken together, our results suggest that in healthy term placentas *MAOA* mRNA levels may serve as a reasonable proxy for MAO-A catalytic capacity, while this regulatory coupling is disrupted under maternal metabolic stress, rendering transcript levels an unreliable proxy for catalytic activity. These findings have implications for interpreting studies that rely solely on *MAOA* expression as a surrogate for enzyme function under pathophysiological conditions.

Key strengths of our study include a well-defined cohort of participants, consisting of full-term (>37 weeks) births by planned cesarean section. This minimized potential confounding effects of gestational age and mode of birth, and ensured a consistent interval between birth and tissue collection. An additional strength is the use of a robust, optimized fluorimetric assay that allowed thorough kinetic characterization across a relatively large, well-balanced sample. The study included approximately equal numbers of participants across four metabolic groups, distributed evenly across experiments, reducing batch effects and strengthening the reliability of group comparisons. Although vaginal births were not included, previous studies have reported no differences in placental MAO activity between cesarean and vaginal births (20), suggesting that our findings are likely representative.

Several limitations of the study should be acknowledged. First, its observational design precludes conclusions about causality between maternal metabolic disorders and alterations in placental MAO-A kinetics. Furthermore, the possible molecular mechanisms underlying the observed alterations in MAO-A substrate affinity in placentas from pregnancies complicated by GDM were not addressed and warrant investigation in future experimental studies. Another limitation is the lack of data linking observed changes in MAO-A kinetics to downstream consequences, such as serotonin levels. Serotonin is difficult to detect in human term placenta, likely due to its rapid degradation (12). Future studies should integrate measurements of placental MAO-A activity with assessments of serotonin and its metabolite levels in the placenta, as well as in maternal and fetal circulation to clarify the downstream impact of altered MAO-A kinetics. Placental tissue was analyzed at term only, precluding evaluation of possible gestational age–dependent changes. MAO activity was measured under *in vitro* conditions using kynuramine as a model substrate, which may not fully reflect the complex *in vivo* placental environment or substrate-specific kinetics. Finally, sex-specific analyses were not performed. In mice, a maternal high-fat diet reduced *MAOA* mRNA levels in female placentas but increased serotonin catabolism in male placentas, supporting sex-specific effects of an obesogenic environment (59). Studies in humans have similarly reported sex-specific changes in placental fatty acid uptake and metabolism in response to maternal metabolic status (60, 61). Therefore, future studies are needed to clarify whether fetal sex modifies placental MAO-A regulation under metabolically compromised conditions.

In conclusion, we developed a microplate-based fluorimetric assay that enables high-throughput assessment of MAO kinetics in human term placenta. Pharmacological experiments identified MAO-A as the predominant catalytically active MAO isoform in this organ. Maternal OWO was not associated with changes in placental MAO-A kinetic parameters, suggesting preserved placental monoamine catabolism. GDM was associated with a modest increase in K_m_ without changes in catalytic capacity, indicating reduced substrate affinity and possible subtle alterations in placental monoamine catabolism. The positive correlation between placental *MAOA* mRNA levels and MAO-A catalytic capacity observed in metabolically healthy pregnancies was absent in metabolically compromised pregnancies, suggesting altered regulatory mechanisms under conditions of metabolic stress. These findings highlight the complex regulation of placental monoamine catabolism and underscore the utility of the standardized kinetic assay for future investigations of MAO-A in placental physiology and pregnancy complications.

## Data Availability

The original contributions presented in the study are included in the article. Further inquiries can be directed to the corresponding author.
